# Response of common bean (*Phaseolus vulgaris* L.) to nitrogen, phosphorus and rhizobia inoculation across variable soils in Zimbabwe

**DOI:** 10.1016/j.agee.2018.08.010

**Published:** 2018-11-01

**Authors:** Vongai Chekanai, Regis Chikowo, Bernard Vanlauwe

**Affiliations:** a Crop Science Department, University of Zimbabwe, Box MP167, Mt. Pleasant, Harare, Zimbabwe; b Plant Soil and Microbial Sciences Department, Michigan State University, East Lansing, MI, 48824, USA; c International Institute of Tropical Agriculture (IITA), N2Africa, ICIPE, Duduville, Nairobi, Kenya

**Keywords:** Common bean, Rhizobia inoculation, Nitrogen, Phosphorus, Smallholder farms

## Abstract

Common bean is an important crop with potential to curb malnutrition in poor Sub-Saharan African populations. Yields of common bean (*Phaseolus vulgaris* L.) are, however poor, limited by low soil phosphorus (P), nitrogen (N) and poor biological N_2_-fixation. On-farm experiments were carried out to study the effect of N, P and rhizobia inoculation on common bean yield and yield components during the 2014/2015 and 2015/2016 cropping seasons in Eastern Zimbabwe. Experiments were conducted on five farmers’ fields located in two agroecologies; three fields were considered to be degraded with soil organic carbon (SOC) < 4 g kg^−1^ and available P < 6 mg kg^−1^, while the two non–degraded sites had SOC > 7 g kg^−1^ and available P > 15 mg kg^−1^. Two common bean varieties (Gloria and NUA45) were tested in a split-plot arranged in randomized complete block design. The main plot factor was the combination of N (0 and 40 kg ha^−1^) and P (0 and 20 kg ha^−1^), and the sub-plot factors were variety (Gloria and NUA 45) and inoculation with *Rhizobium tropici* strain CIAT899 (+/− inoculum). At planting, both N and P were applied at 20 kg ha^−1^, with an additional 20 kg ha^−1^ N top dressing applied at flowering. Analysis of variance indicated common bean did not respond to rhizobia inoculation (P > 0.05) whilst P significantly increased the number of nodules and active nodules per plant (P < 0.001), and grain yield. Application of 40 kg ha^−1^ N significantly increased the number of pods per plant, number of seeds per pod, and grain yields. A significant NP interaction was only observed on grain yield for non-degraded soils. Co-application of N and P in non-degraded sites increased grain yields from 0.27 to 1.48 Mg ha^−1^during the first season and from 0.37 to 2.09 Mg ha^−1^during the second season. On degraded sites, NP application resulted in uninspiring grain yield gains of 0.09 to 0.19 Mg ha^−1^ during the first season, and from 0.16 to 0.28 Mg ha^−1^ in the second season. In general, effects of N or P were not significantly different, suggesting that farmers could invest in either of these nutrients for increased common bean grain yields. Strategically, P investments would be more logical as residual P effects to rotational cereals improve overall cropping system performance. The response of common bean to inoculation in Zimbabwe still needs to be widely investigated for these and other varieties.

## Highlights

• N and P had comparable effects on common bean productivity.
• Main common bean varieties used in Zimbabwe do not respond to inoculation.
• Investments in N and P on acutely degraded soils does not result in common bean yield gains.

## 1 Introduction

Common bean (*Phaseolus vulgaris* L.) is an important grain legume throughout the world providing a source of protein, dietary fibre, starch and minerals such as potassium, thiamine, vitamin B6 and folic acid in diets affordable by the poor (Garden-Robinson and McNeal, 2013). Edaphic and environmental factors that constrain bean production in most areas where the crop is grown include nitrogen and phosphorus deficiency, soil acidity (including aluminium and manganese toxicity) and drought (Bationo, 2004; Amede et al., 2004). Due to these factors, current bean yields in Southern Africa average only 0.6 Mg ha^−1^compared to attainable yields of >1.5 Mg ha^−1^ (Chianu et al., 2011).

A hallmark trait of agriculturally-useful legumes is their symbiosis with rhizobia bacteria which fix atmospheric nitrogen (N2) within root nodules and make it available to the host plant (Giller, 2001). N_2_-fixation is dependent upon factors such as adequate supply of micro and macro nutrients which are needed by both the plant and the rhizobia bacteria (Musandu and Ogendo, 2001). Although common bean has good potential for N_2_fixation, it is reported to have the lowest N2-fixation rate among the most widely grown grain legumes (Giller, 1990; Martinez-Romero, 2003). Inoculation of common bean with rhizobia strains has been shown to be beneficial in increasing nodulation thereby enhancing biological nitrogen fixation, but in many cases, effective nodulation is also affected by competition from high populations of competitive but ineffective native rhizobia (Giller, 2001). Plant available P in soils is particularly important for sufficient nodulation and N_2_-fixation. Phosphorus is an essential nutrient for various metabolic processes such as photosynthesis, respiration, and signal transduction, among others. Phosphorus application for on common bean consistently showed a positive response on yield, shoot dry matter and number of pods (Turuko and Mohammed, 2014; Fageria and Baligar, 2016). Nodule number, weight and volume also increased with the addition of P, indicating more effective N_2_-fixation (Singh et al., 2008; Rifat et al., 2008). Nitrogen fixation in common bean has also been established to be more affected by P deficiency than in other legume crops such as soybean (Fageria and Baligar, 2016), with Gidago et al. (2011) reporting adequate P rates for maximum yield and yield attributes as high as 40 kg ha^−1^.

While most grain legumes only require ‘starter’ N to initiate early growth before the N2-fixing symbiosis is established, in Zimbabwe, market-oriented farmers on sandy soils often apply additional top-dressing N fertilizer for high yields. In Brazil, Henson and Bliss (1991) noted that there was a positive yield response when N was applied to common bean plants grown on N-poor soils. They recommended application of N during vegetative growth as the best management system. However, large amounts of plant-available N tend to inhibit rates of N2-fixation (Giller, 2001), suggesting that farmers could forfeit the ecological benefits of integrating common bean in their cropping system.

In this study, our intention was to build on the existing knowledge from other studies on common bean nutrition. Our objectives were to: (1) investigate the effect of N, P and rhizobia inoculants on productivity of common beans across different soils and in two agro-ecological zones where common bean is typically grown on smallholder farms, and (2) provide an alternative fertilization strategy for common bean in the context of common crop sequences.

## 2 Materials and methods

### 2.1 Study sites and field sites characterization

Five on-farm experiments were implemented in two smallholder farming communities of Domboshawa (17°36´S, 31°10´E) and Murehwa (17°45´S, 31°34´E) in Eastern Zimbabwe, during the 2014/15 and the 2015/2016 cropping seasons. We intended to set up experiments on at least two farms, with soils representative of degraded and non-degraded soil fertility conditions in each of the two farming areas. We therefore initially took soil samples and analysed for SOC content for 20 field sites in each of the farming communities. Soil organic carbon is known to be a robust index for soil health (e.g. Nandwa, 2001). Prospective field sites were grouped into those that had SOC < 4 g kg^−1^, and those that had SOC > 7 mg kg^−1^. We subsequently used this information to narrow our field selection to the five field sites we used (Table 1).

**Table 1:** Physical and chemical soil properties taken from experimental sites (1–20 cm) in Domboshava and Murehwa in Eastern Zimbabwe.

Site	Farmer	Fertility	Clay	Sand	SOC	Total N	Available P (mg kg^−1^)	Soil pH (water 1:10)	Exchangeable bases (cmol_(+)_ kg^−1^)		
			(g kg^−1^)						Ca	Mg	K
Domboshava	Chawonza	High	120	850	7.9	0.7	16.3	5.7	5.52	1.05	0.1
	Kaviya	Low	100	800	3.2	0.5	5.90	4.7	1.26	1.10	0.1
Murehwa	Madziva	High	100	780	7.4	0.7	15.2	5.8	5.31	1.37	0.3
	Faro	Low	80	880	3.7	0.5	5.80	5.3	1.18	1.40	0.3
	Marimo	Low	80	760	3.9	0.5	5.90	4.9	1.80	2.10	0.1

Domboshawa is located in natural agro-ecological region IIa (NR IIa) that receives reliable rainfall averaging 900 mm year^−1^, while Murehwa is located in a relatively drier NR IIb, receiving about 750 mm year^−1^ annually. Rainfall is unimodal, and is received from November to April. Zimbabwe is divided into five natural agro-ecological regions (NR I-V) with NR I having the most favourable conditions, receiving >1000 mm rainfall while semi-arid NR V receives annual average rainfall of <500 mm (Vincent and Thomas, 1960). Rain-fed common bean production is mainly confined to NR II, that is further sub-divided into ‘a’ and ‘b’ zones for finer targeting of crops that are sensitive to temperature or rainfall amounts. In these areas, farmers generally grow improved common bean varieties as sole crops while local landraces are often intercropped with maize. Farms average about 3 ha, with maize occupying at least 50% of the cropped lands. Other major grain legume crops in the study sites include cowpea and groundnut.

Before the cropping season, composite soil samples consisting of five sub-samples were collected from the plough layer (0–20 cm depth) along the fields’ diagonal and bulked for each of the five sites. The soil samples were air-dried, and those for total N, available P, extractable bases analysis were passed through 2 mm sieve, while those for SOC analysis were passed through a 0.5 mm sieve. Total SOC was determined by the modified Walkley-Black) (Okalebo et al., 2002), while total N and available P were determined by the micro-Kjeldahl and Olsen methods, respectively (Anderson and Ingram, 1993). Extractable bases (K, Mg and Ca) were extracted using ammonium acetate. Potassium (K) was determined by flame photometry, and Ca and Mg concentrations were determined by atomic absorption spectrophotometry. Soil pH was determined using the 1:10 water method and soil texture was determined using the hydrometer method (Gee and Bauder, 1986).

### 2.2 Experimental design and management

Experimental treatments were designed to explore the interaction of nitrogen (N), phosphorus (P) and rhizobia inoculation (+I) on two common bean varieties (Gloria and NUA45) using site as a random factor. In each field, the experiments were laid out in a split plot arranged in randomized complete block design replicated in three blocks. Main plots were nested within the blocks and sub-plots were nested within the main plots. The main plot treatments were fertilizer management [no fertilizer, N, P or NP], and subplots were randomly assigned to +/- inoculation and variety, resulting in the following treatments:

i) Control (no fertilizer or rhizobia added),
ii) NP + I [ammonium nitrate (34.5% N) + single super phosphate + inoculation],
iii) NP (ammonium nitrate + single super phosphate),
iv) N + I (ammonium nitrate + inoculation),
v) P + I (single super phosphate + inoculation),
vi) N (ammonium nitrate only),
vii) P (single super phosphate only), and
viii) +I (inoculation only).

Two improved varieties, Gloria and NUA 45 that are both maintained by Zimbabwe Crop Breeding Institute and locally available on the market in Zimbabwe, were used. The varieties are both determinate and take approximately 90–100 days to reach physiological maturity. A rhizobia inoculum (*Rhizobium tropici* strain CIAT899) obtained from a local commercial manufacturer (Soil Productivity Research Laboratory, Marondera) was used to inoculate the bean seed prior to sowing, using a rate of 100 g inoculum per 25 kg of seed as recommended by the manufacturer. Phosphorus was applied at 20 kg ha^−1^ P at planting, while N was applied in two splits, 20 kg ha^−1^ at planting plus an additional 20 kg ha^−1^ N applied at flowering stage, for a total N rate of 40 kg ha^−1^. All treatments that had P applied also received 12 kg ha^−1^ sulphur since single super phosphate also contains 5%. Sulphur is generally deficient on these sandy soils.

**Fig. 1:**
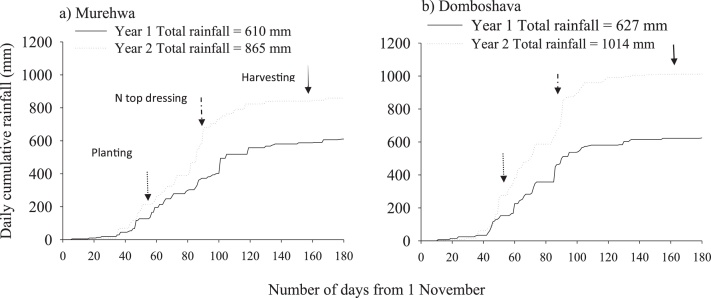
Cumulative daily rainfall for 2015/2014 and 2015/2016 cropping seasons in Domboshava and Murehwa, Zimbabwe. Arrows indicate when major agronomic practices were implemented.

Land preparation was done using ox-drawn plough and plots were established at 4 m × 4 m size. Planting for the first season was done on the 28 and 29th December 2014 during the 2014/15 cropping season, and on the 1st and 2nd of January 2016 for the 2015/2016 cropping season. Planting was done about 6 weeks after the onset of the rains to prevent physiological maturity from coinciding with the normally high rainfall during February. Common bean was planted using an inter-row spacing of 45 cm and intra- row spacing of 10 cm for a plant population of 222,000 plants ha^−1^. Prior laboratory seed germination tests had established a nearly 100% seed viability for both varieties, which was also achieved in the field. The plots were kept weed-free by hand-hoeing throughout the growing seasons. Cumulative daily rainfall for the two seasons and major agronomic events are shown in Fig. 1.

### 2.3 Determination of nodulation and dry biomass

At 6–7 weeks after germination, destructive sampling of plants was done within a 1 m × 1 m quadrat in each plot, excluding the border rows and the net plot. Ten common bean plants were randomly sampled from the uprooted plants, carefully washed in water to remove excess soil. The number of nodules per plant was determined by counting all nodules on each of the 10 plants and computing the average. Number of active nodules was determined by cutting nodules on each of the 10 plants and observing the colour inside the nodule. Active nodules were identified by a pink to reddish internal colour. Fresh weight was then determined by immediately weighing all the uprooted plants using a digital scale. The samples were then oven-dried at 65 0C for 3 days and weighed to determine dry biomass.

### 2.4 Determination of yield components and grain yields

At physiological maturity, all the plants in 1.8 m × 1.8 m net plots (4 rows × 1.8 m long) were cut at soil level and heaped at the centre of the plot. Random sampling of 10 plants was done and all the pods on each plant were counted and recorded to determine the number of pod per plant. A sub-sample of the pods was used to determine the number of seeds per pod. Later, all pods from the net plot were harvested into perforated harvest bags, sun-dried for 14 days and threshed. The grain was then weighed and grain moisture content determined using a John Deere SW moisture meter. Yields reported here are adjusted to 10% moisture content.

### 2.5 Statistical analysis

The number of nodules and active nodules per plant, number of pods per plant and number of seeds per pod were transformed using quantile normalization and subjected to analysis of variance (ANOVA) using R Version i3863.32. Table 2 shows an example of ANOVA table for grain yield obtained using a split plot model at one site, with different errors for main plots and subplots. The magnitude of the difference in all variables measured from degraded and non-degraded sites was huge; therefore data from these soil fertility domains was analysed separately with site considered as a random factor.

**Table 2:** ANOVA table for the split plot design used in this study that had fertilizer management (N and P) as main plot, and variety and inoculation as subplots. This example shows ANOVA for grain yield at a non-degraded Chawonza site for Year 1.

Source of variation	Degrees of Freedom	Sum of Squares	Mean Square	F value	Pr (>F)
Block	2	1027661	513830	2.07	0.207
Nitrogen	1	1572407	1572407	6.35	0.045
Phosphorus	1	3674625	3674625	14.83	0.008
Nitrogen x phosphorus	1	3056317	3056317	12.33	0.013
*Main plot error*	6	1486759	247793	0.42	
Variety	1	1110	1110	0.00	0.966
Inoculation	1	753,783	753,783	1.28	0.269
Nitrogen x variety	1	309,801	309,801	0.53	0.475
Phosphorus x variety	1	62,681	62,681	0.11	0.747
Nitrogen x inoculation	1	216,471	216,471	0.37	0.550
Phosphorus x inoculation	1	34,956	34,956	0.06	0.810
Variety x inoculation	1	135,835	135,835	0.23	0.635
Nitrogen x phosphorus x variety	1	552,886	552,886	0.94	0.342
Nitrogen x phosphorus x inoculation	1	198,847	198,847	0.34	0.566
Nitrogen x variety x inoculation	1	1,253,599	1,253,599	2.13	0.157
Phosphorus x variety x inoculation	1	25,633	25,633	0.04	0.836
Nitrogen x phosphorus x variety x inoculation	1	898,174	898,174	1.53	0.229
*Sub plot error*	24	14122590	588441		
Total	47	29384135			

## 3 Results

### 3.1 Soil characterization

All sites had low clay content, ranging from 8–12%,while two sites that had poor SOC content of less than 0.4% were concomitantly acidic (pH < 5.5) and acutely deficient in available P (Table 1). These infertile fields are subsequently referred to as “degraded’ and the remainder of the sites as “non-degraded”. We evaluated the response of common bean to management separately in the degraded and non-degraded fields due to these distinct differences in soil fertility.

### 3.2 N, P and + I effects on nodulation and pod loading

There was no significant variety effect; therefore, data presentation is at the crop level throughout the paper. In degraded soils with SOC < 0.4%, none of the tested factors significantly influenced nodulation and pod loading. During the first season, analysis of variance showed significant differences in the number of nodules per plant when 20 kg ha^−1^ P was added (p = 0.006). The number of nodules per plant increased from three in the control to eight in the P treatment (Table 3). Similar results were observed during the second season where P application significantly increased (p < 0.001) number of nodules per plant from four in the control to nine in the P treatment (Table 3). Phosphorus also significantly (p < 0.001) increased the number of active nodules per plant for both seasons, from two in the control to a maximum of six in the P treatment during Year 1 (Table 3). Co-application of phosphorus and rhizobia (P + I) did not result in significant increases in the number of active nodules during both seasons.

**Table 3:** Common bean nodulation as influenced by nitrogen, phosphorus and rhizobia inoculation in fairly fertile soils with >0.7% SOC. Mean separation was done using transformed values that are outside parenthesis. Values with different letter(s) are significantly different at 5% probability.

Treatments	Number nodule plant^−1^		Number of active nodules plant^−1^	
	Year 1	Year 2	Year 1	Year 2
Control	1 ± 0.8^a^ (3)	2 ± 0.8^a^ (4)	1 ± 0.6^a^ (2)	3 ± 0.7^a^ (3)
+Inoculation	1 ± 1.0^a^ (5)	2 ± 1.0^a^ (5)	1 ± 0.8^a^ (3)	3 ± 0.9^a^ (4)
+Nitrogen	3 ± 1.4^a^ (4)	2 ± 1.0^a^ (4)	3 ± 1.4^a^ (2)	3 ± 0.9^a^(3)
+Phosphorus	13 ± 1.4^b^ (8)	11 ± 1.0^b^ (9)	11 ± 1.4^b^(6)	9 ± 0.9^b^ (8)
+Phosphorus + Inoculation	13 ± 2.5^b^ (8)	11 ± 1.4^b^ (9)	11 ± 1.6^b^ (6)	9 ± 1.2^b^ (8)
+Nitrogen + Inoculation	3 ± 2.5^a^ (4)	2 ± 1.4^a^ (4)	3 ± 1.6^a^ (3)	3 ± 1.2^a^ (4)
+Nitrogen + Phosphorus	11 ± 2.0^b^ (6)	10 ± 1.3^b^ (8)	9 ± 1.9^b^ (5)	9 ± 1.2^b^ (7)
+Nitrogen + Phosphorus + Inoculation	11 ± 3.0^b^ (6)	11 ± 2.0^b^ (8)	9 ± 2.2^b^ (5)	9 ± 1.8^b^ (7)
CV %	103	103	135	84

Analysis of variance showed that pod numbers were significantly increased by 40 kg ha^−1^ N in the first season (p = 0.02) and the second season (p = 0.003). Increases in pod number from four in the control to eight in the N only treatment were observed in the first season, and eight to 11 during the second season (Table 4). The number of seeds per pod were significantly (p = 0.03) increased by the addition of N for both seasons. In all cases, N application more than doubled the number of pods per plant and number of seeds per pod (Table 4).

**Table 4:** Influence of nitrogen, phosphorus and rhizobia inoculation on number of pods per plant and number of seeds per pod in soils with >0.7% SOC. Mean separation was done using transformed values that are outside parenthesis. Values with different letter(s) are significantly different at 5% probability.

Treatments	Number pods plant^−1^		Number of seeds pod^−1^	
	Year 1	Year 2	Year 1	Year 2
Control	3 ± 0.2^a^ (4)	9 ± 0.4^a^ (8)	2 ± 0.1^a^ (2)	2 ± 0.1^a^ (2)
+Inoculation	3 ± 0.3^a^ (4)	9 ± 0.5^a^ (8)	2 ± 0.1^a^ (2)	2 ± 0.1^a^ (2)
+Phosphorus	4 ± 0.4^a^ (4)	8 ± 0.6^a^ (9)	4 ± 0.1^b^ (4)	4 ± 0.1^b^ (4)
+Nitrogen	9 ± 0.4^b^ (8)	12 ± 0.6^b^ (11)	4 ± 0.1^b^ (4)	4 ± 0.1^b^ (4)
+Phosphorus + Inoculation	3 ± 0.5^a^ (4)	8 ± 0.7^a^ (8)	4 ± 0.2^b^ (4)	4 ± 0.2^b^ (4)
+Nitrogen + Inoculation	9 ± 0.5^b^ (8)	12 ± 0.7^b^ (11)	4 ± 0.2^b^ (4)	4 ± 0.2^b^ (4)
+Nitrogen + Phosphorus	9 ± 0.6^b^ (8)	12 ± 0.8^b^ (11)	4 ± 0.2^b^ (4)	4 ± 0.2^b^(4)
+Nitrogen + Phosphorus + Inoculation	10 ± 0.8^b^ (9)	13 ± 1.0^b^ (12)	4 ± 0.3^b^ (4)	4 ± 0.3^b^ (4)
CV %	53	40	50	37

### 3.3 N, P and + I effects on biomass and grain yields

**Fig. 2:**
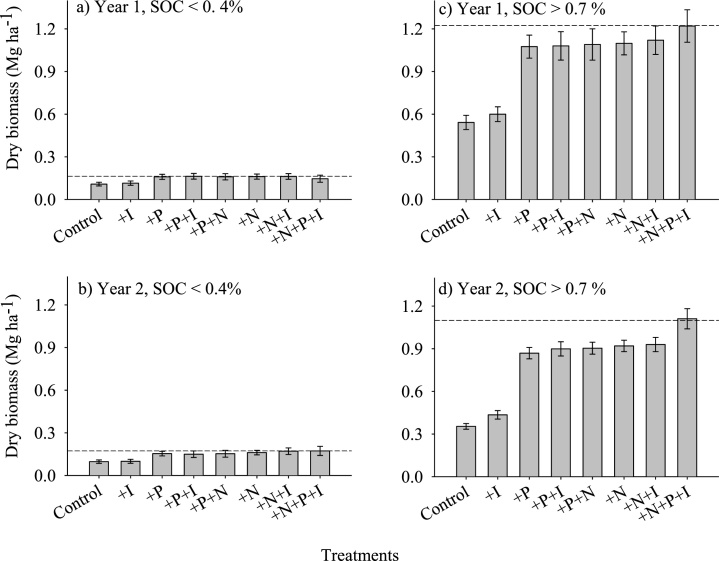
Common bean dry biomass as influenced by nitrogen, phosphorus and rhizobia inoculation during the 2014/2015 and 2015/2016 cropping seasons in Eastern Zimbabwe.

Common bean dry biomass was significantly increased by application of N, P and NP in both degraded and non-degraded soils, but biomass was a maximum of only 0.17 Mg ha^−1^ under degraded soils compared 1.2 Mg ha^−1^ for non-degraded soils (Fig. 2). There was no response to rhizobia inoculation on degraded soils for both years, while only marginal biomass gains were observed on non-degraded soils. In all cases, co-application of N and P did not result in biomass yield differences from the N or P only treatments.

**Fig. 3:**
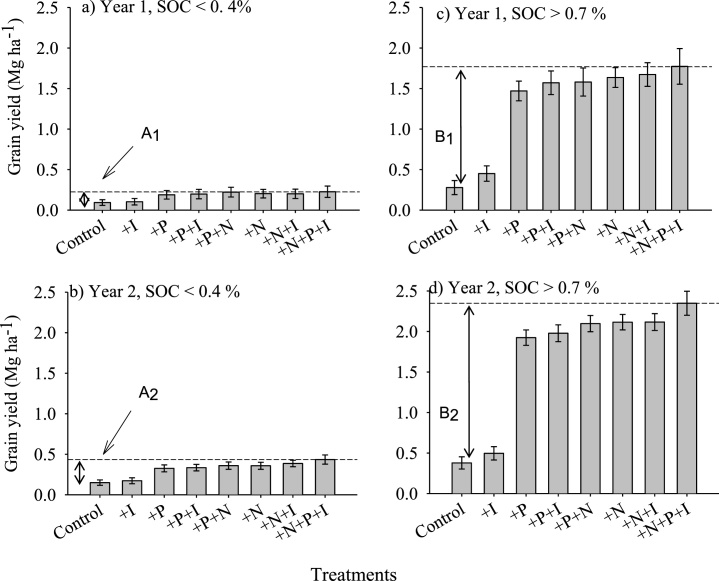
Common bean grain yields as influenced by nitrogen, phosphorus and rhizobia inoculation (+I) during the 2014/2015 and 2015/2016 cropping seasons in Eastern Zimbabwe. A_1_ and A_2_ show yield gaps associated with degraded soils while B_1_ and B_2_ are common bean yield gaps on non-degraded soils.

Application of N or P equally significantly increased common bean grain yields, but there were generally no benefits of co-application of N and P for both degraded and non-degraded soils (Fig. 3). The exception was during Year 2 when NP application resulted in larger yields under non-degraded soils. Under degraded soils, grain yields without any fertilizer were a paltry 0.27 Mg ha^−1^ and 0.37 Mg ha^−1^, for Years 1 and 2, respectively, and only a maximum of 0.43 Mg ha^−1^ with NPI application during Year 2. For the non-degraded soils, grain yields increased from 0.27 to 1.77 Mg ha^−1^ when NPI was applied during Year 1, and from 0.37 to 2.3 Mg ha^−1^ with NPI application. Inoculation with rhizobia only did not result in significant yield increases although grain increased in PI, NI and NPI treatments. These results indicate that base yields for non-degraded soils are comparable or larger than yields obtained with NP fertilization on degraded soils. Based on the limited nutrient inputs used and without liming to ameliorate soil pH, the practical exploitable yields gaps on degraded soils were only 0.13 and 0.28 Mg ha^−1^ (A_1_ and A_2_), while the potential benefits of fertilizing non-degraded soils were 1.5 and 1.93 Mg ha^−1^ (B_1_ and B_2_) (Fig. 3). Inoculation with rhizobia only or in combination with N and P did not influence common bean productivity for both varieties.

## 4 Discussion

### 4.1 Common bean response to management - the soil fertility factor

While common bean originated from regions with moderately fertile soils, globally, the cultivation of common bean by smallholder farmers on degraded soils has contributed to poor productivity (Beebe et al., 2012). In many farming communities in Africa, farmers often preferentially allocate cereal crops to more fertile fields on their farms, with legumes relegated to soils with multiple constraints (Zingore et al., 2007). If soils are not severely depleted in nutrients, this strategy may ensure successful production of both cereals and legumes in cases where legumes’ ecological capabilities are sufficient to overcome the soil infertility hurdle. This is often the case with cowpea that is drought tolerant and adapted to stressful environments where many crops fail to grow well (Abaidoo et al., 2007; Carvalho et al., 2017). However, common bean has little tolerance to low soil fertility (Singh et al., 2003). Allocating common bean to more marginal fields, as is often the case with cowpea, will not produce the same result. In our study, the performance of the two varieties we tested was consistently poor at three sites that had SOC < 0.4% and over two seasons. Application of both N and P did not result in any significant yield gains on these soils, this contrasted sharply with soils that had SOC > 0.7% (Fig. 3). These results strongly suggest multiple soil fertility limitations for common bean production that cannot be addressed by application of only N and P fertilizers that farmers often use in the study sites. Other than the poor SOC and available P contents, the degraded sites were also acidic (Table 1). Non-responsive soils such as these have been described earlier (Vanlauwe et al., 2015). They develop over time because of several factors, including nutrient mining crop production practices and soil erosion. Lack of adequate organic soil amendments that add basic cations and ameliorate soil pH compounds the problem (Mtambanengwe and Mapfumo, 2005), with farmers ultimately abandoning such fields.

Inoculating common bean with rhizobia gave no significant increase in nodulation, biomass or grain production (Fig. 3). Rebeschini et al. (2014) also found that inoculation of beans with *R. Tropici* gave no positive response. Hungria and Vargas (2000) also reported that inoculation with rhizobia in field experiments rarely increases yield of beans. The poor response of common bean to rhizobia inoculation observed in this study could be attributed to failure by the strains used to adapt to the harsh abiotic conditions. In other studies, abundant native and ineffective rhizobia strains in the soils competed with the introduced inoculum to form nodules, while only certain rhizobia strains had the ability to fix N in specific cultivars (Valverde et al., 2003). The rhizobia bacteria is highly sensitive to moisture stress and requires high amount of photosynthate and P. The interaction of these factors and the environment reduces the capability of most common bean cultivars to fix N in the tropics and subtropics (Fageria et al., 2014).

Phosphorus fertilization significantly increased nodulation and number of active nodules, but only for non-degraded soils (Table 3). Strong increases of nodulation with P fertilizer have frequently been found when there is little soil P available (Giller et al., 1998; Leidi and Rodríguez-Navarro, 2000; Tang et al., 2001). Phosphorus fertilization improves early root formation facilitating increased nodulation and enhanced common bean productivity. Nitrogen had a significant effect on pod loading, number of seeds per pod, and yields (Table 4; Fig. 3). Da Silva et al. (1993) reported increased common bean grain yields with application of N in N-deficient soils. Top-dressing common bean with N has also been reported to increase common bean yield (Arf et al., 2011; Argaw and Akuma, 2015). Chidi et al. (2002), however, reported that common bean response to N varies with cultivars and environmental factors.

### 4.2 Common bean fertilization strategy

Application of N or P had comparable effects on common bean productivity, with no clear benefits of co-application of these nutrients in most of the cases (Fig. 3). Market–oriented smallholder farmers in Zimbabwe regularly fertilize common bean with N on sandy soils. With a cropping systems improvement objective, we content that it would be prudent to prioritize P fertilization to common bean and benefit from residual P effects for cereal crops grown in sequence (Rurangwa et al., 2017). Generally, P recovery rarely exceeded 10% when single super phosphate was applied to soyabean on sandy soils in Zimbabwe (Zingore et al., 2008).

## 5 Conclusions

Application of N or P had equal magnitude of increasing common bean grain yields with no significant benefits of adding both elements. This result is important as farmers in the study area regularly invest more in N fertilizers for common bean than in P. With well documented P residual benefits to crops in rotations, direct P fertilization to common bean is expected to improve cropping system performance. This study also established that the improved common bean varieties that are currently on the market did not respond to rhizobia inoculum currently marketed in Zimbabwe. We also confirmed the existence of degraded non-responsive soils. While some ‘wonder’ legumes such as cowpea can be successfully grown on infertile soils, attempts to grow common bean on such soils results in very low yields.
